# Craniofacial features as assessed by lateral cephalometric measurements in children with Down syndrome

**DOI:** 10.1186/s40510-016-0148-7

**Published:** 2016-11-07

**Authors:** Veerasathpurush Allareddy, Nicholas Ching, Eric A. Macklin, Lauren Voelz, Gil Weintraub, Emily Davidson, Lisa Albers Prock, Dennis Rosen, Richard Brunn, Brian G. Skotko

**Affiliations:** 1 Department of Orthodontics, College of Dentistry and Dental Clinics, The University of Iowa, Iowa City, IA USA; 2 Children’s Dentistry, El Cerrito, CA USA; 3 Biostatistics Center, Massachusetts General Hospital and Harvard Medical School, Boston, MA USA; 4 Department of Medicine, Down Syndrome Program, Division of Developmental Medicine, Boston Children’s Hospital, Boston, MA USA; 5 David Geffen School of Medicine at the University of California, Los Angeles, CA USA; 6 Department of Pediatrics, Harvard Medical School, Boston, MA USA; 7 Department of Medicine, Division of Respiratory Diseases, Boston Children’s Hospital, Boston, MA USA; 8 Department of Dentistry, Boston Children’s Hospital, Boston, MA USA; 9 Department of Pediatrics, Down Syndrome Program, Division of Medical Genetics, Massachusetts General Hospital, Boston, MA USA

## Abstract

**Objective:**

The objective of the present study is to examine the craniofacial development of patients with Down syndrome (DS) and compare them with a neurotypical population.

**Methods:**

This study is a cross-sectional analysis of lateral cephalometric radiographs of participants with DS. The study population consisted of children and young adults with DS aged 3–25 years. Cephalometric data were summarized by age and sex. Raw and normalized z-scores were computed. One-sample *t* tests were used to test whether mean z-scores differed from zero. The demographic characteristics between those with or without lateral cephalograms among all study participants were compared by Fisher’s exact tests.

**Results:**

The study sample comprised of 27 participants with DS. Study subjects demonstrated a class III skeletal pattern. This was more pronounced in the older age groups as compared to younger age groups. Subjects also had an increased proportionate lower anterior face height to total facial height compared to normative standards. Gonial angles, mandibular plane angles, and airway measurements increased with age.

**Conclusions:**

Patients with Down syndrome present typically with class III skeletal pattern and long lower anterior facial heights. In patients with Down syndrome, comprehensive phase of orthodontic treatment may be best initiated following cessation of growth.

## Background

Down syndrome (DS) is a chromosomal condition occurring in about 1 in every 792 live births [1, 2]. Multiple prior reports have described the craniofacial morphological features of DS [3–12], which include a short and flat cranial base, maxillary hypoplasia, midface retrusion, skeletal class III pattern, variations in mandible (including normal or reduced gonial angle; normal, retruded, or prognathic mandible; and variations in mandibular plane angle), skeletal anterior open bites, long lower anterior facial heights, and proclination of maxillary and mandibular incisors. Whether the maxillary hypoplasia is due to reduction in overall dimensions of the head is unclear and has not been well described in previous studies. Furthermore, there is no uniform consensus on their mandibular morphology. Some studies have shown their mandibles to be small; while others have shown mandibles to be similar to neurotypical controls [9, 11–13].

The objective of the present study is to examine the craniofacial development of patients with DS and compare them with a neurotypical population obtained from the Iowa longitudinal growth study. This study tests the hypothesis that individuals with DS demonstrate a different craniofacial pattern, measurable on a lateral cephalometric radiograph, when compared to neurotypical controls.

## Methods

### Institutional review board approval and informed consent

The study purpose, goals, and objectives were explained in detail to all study participants and their caregivers; informed consent was obtained from them prior to clinical examinations and exposure of lateral cephalometric radiographs. The present study was approved as protocol 10-03-0092 by the Human Participants Protection Office of Boston Children’s Hospital (BCH).

### Study design and study population

The present study is a cross-sectional analysis of lateral cephalometric radiographs of participants with DS. The study population consisted of children and young adults, ages 3–25 years, who attended the Down Syndrome Program at BCH. This study was nested in a larger study examining obstructive sleep apnea in patients with DS; as such, all children with DS who already had a polysomnogram with the past 6 months, an adenotonsillectomy, adenoidectomy, or tonsillectomy were excluded from this study.

### Clinical examination

For each study participant, a dental examination was conducted by a dental provider. Two providers were calibrated for the study, and all dental examinations were conducted by these two providers (VA and NC). The dental exams consisted of both extraoral and intraoral assessments. The extraoral assessment consisted of evaluation of each patient’s profile in which the presence of soft tissue maxillary and/or mandibular hypoplasia/retrognathia was observed. The intraoral exam consisted of recording each patient’s centric occlusion with overbite recorded as a percentage of the overlap between the right maxillary and mandibular incisor’s clinical crown (and also in mm) and with the overjet recorded with a ruler as millimeter measurements from the facial surface of the right mandibular incisor to the incisal edge of the right maxillary incisor. Examination of the oropharynx was evaluated by use of the Mallampati scores and Friedman Tonsil Classification System [14, 15]. Mallampati scoring is based on the visibility of the base of the uvula, faucial pillars, and soft palate and is divided into four classes: class 1, full visibility of tonsils, uvula, and soft palate; class 2, visibility of hard and soft palate, upper portion of tonsils and uvula; class 3, soft and hard palate and base of the uvula are visible; and class 4, only hard palate visible [14]. Friedman classification is used to evaluate tonsillar size. Friedman scores are divided into four classes: grade 1+, the tonsils are hidden within the tonsillar pillars; grade 2+, the tonsils extend to the tonsillar pillars; grade 3+, the tonsils extend beyond the pillars but do not reach the midline; and grade 4+, the tonsils extend to, or beyond, the midline [15]. A dental exam was considered complete if all extraoral and intraoral measurements of the examination were performed and completed. This included a repeatable and reliable occlusion.

### Exposure of lateral cephalometric radiographs

After successfully completing the dental examination, a lateral cephalometric radiograph was exposed for each patient. Using the Sirona ORTHOPHOS XG Plus lateral cephalograph system, patients were placed in a seated, upright position. With the patient asked to look into the horizon, the examiner determined the natural head posture in the upright position. In order to obtain a natural head position, the participant’s parent would often participate by facing the participant at their eye level. Each participant was instructed to swallow and then lightly contact the back teeth into maximum intercuspation to bring the mandible into centric occlusion. The participant was asked to breathe normally and, within 10 s of swallowing, the lateral cephalogram was obtained to ensure consistent tongue and mandibular position [16]. The film was taken at end-expiration to control for the effect of lung volume on upper airway size [17]. To ensure a high level of contrast on the cephalograms, radiographic exposure consisted of either 64 kV and 8 mA or 69 kV and 15 mA depending on the amount of the participant’s head and neck soft tissue. A maximum of two cephalograms were exposed. If the participant moved or was unable to be properly positioned, the attempt to obtain a diagnostic cephalogram was aborted and considered incomplete.

### Assessment of lateral cephalometric radiographs

**Fig. 1:**
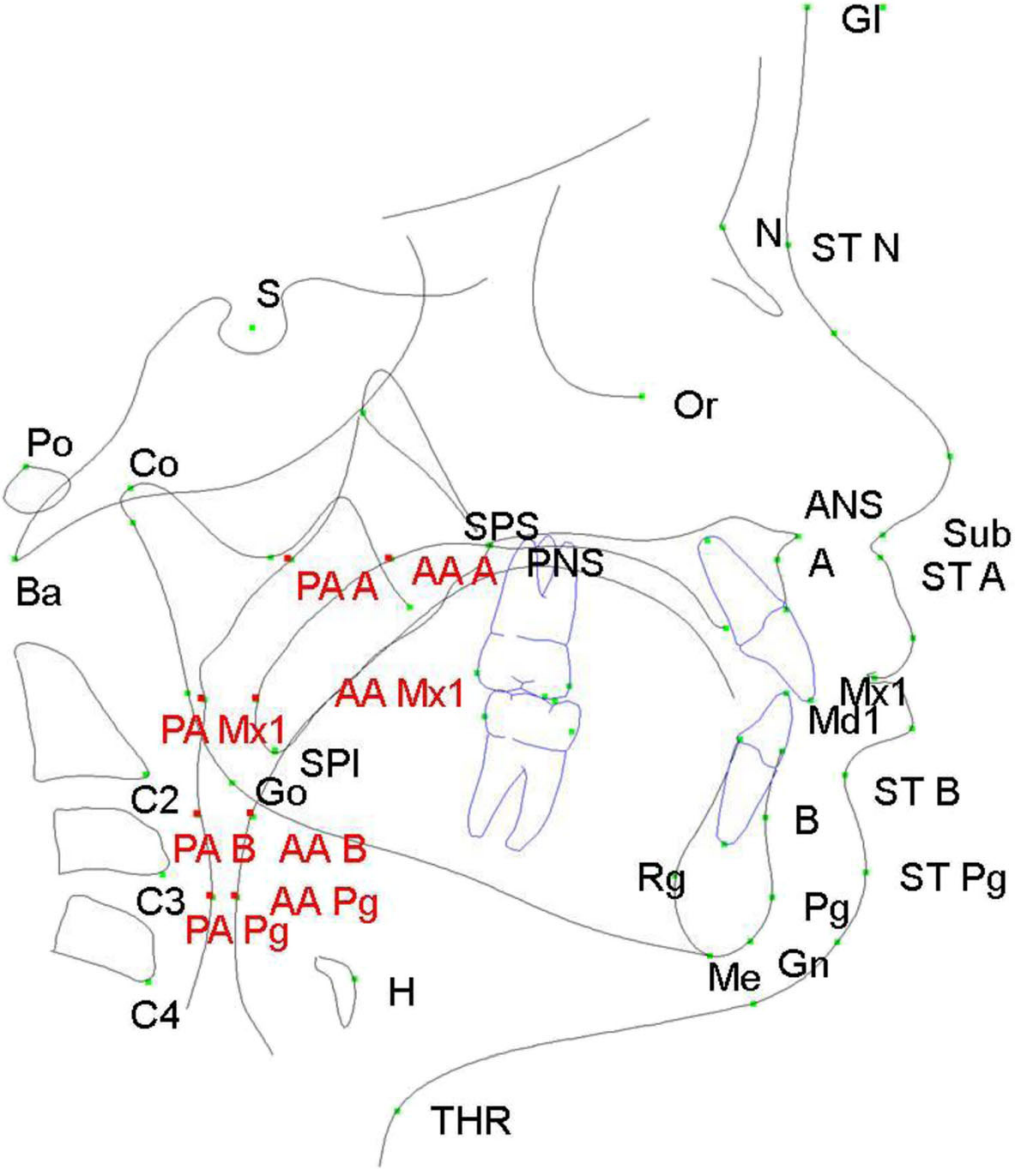
Diagrammatic representation of anatomic landmarks used to identify craniofacial and soft tissue parameters on cephalometric radiographs. Sixty one landmarks and 40 measurements were included in the custom analysis. Airway landmarks are highlighted in *red*. See Table 1 for explanation of abbreviations

Dolphin Imaging software was used to analyze craniofacial landmarks and measurements. With the head oriented in a natural head position, landmarks were placed on the average location of the left and right structures. A custom analysis tool was utilized to evaluate conventional dental and craniofacial structures, such as the maxilla, mandible, and cranial base. Additionally, several soft tissue points and contours of the soft palate, hyoid, pharynx, and hard-tissue points, such as the cervical vertebrae, were also measured (Fig. 1, Tables 1 and 2). Arnett’s true vertical line, which is defined as a line drawn from 8.5 mm away from soft tissue glabella through subnasale, was chosen to minimize participantivity. For images where participants were not in maximum intercuspation, as determined by comparison of the clinical occlusion and radiographic occlusion, dolphin “treatment simulation” was utilized to close the bite around the hinge axis. Thus, the inappropriate measurements (e.g., hyoid) were excluded.

**Table 1:** Abbreviations for anatomical landmarks and reference planes used in the analysis of lateral cephalograms

Landmark	Description	Landmark	Description
S	Sella turcica	C2	Inferior, anterior point of cervical vertebra 2
N	Nasion	C3	Inferior, anterior point of cervical vertebra 3
A	Subspinale	C4	Inferior, anterior point of cervical vertebra 4
B	Supramentale	C5	Inferior, anterior point of cervical vertebra 5
ANS	Anterior nasal spine	ST N	Soft tissue nasion
PNS	Posterior nasal spine	ST A	Soft tissue A point
Ba	Basion	ST B	Soft tissue B point
Or	Orbitale	ST Pg	Soft tissue pogonion
Gn	Gnathion (anatomical)	ST G	Soft tissue gnathion
Go	Gonion	ST M	Soft tissue menton
Me	Menton	ULP	Upper lip point
Po	Porion	LLP	Lower lip point
Pg	Pogonion	MR	Mid ramus
Gl	Glabella (soft tissue)	Sig	Sigmoid notch
Ar	Articulare	PT	Pterygopalatine point
Rg	Retrognathion	Bno	Bridge of nose
H	Anterior, superior point of the hyoid	Tno	Tip of nose
Co	Condylion	Sub	Subnasale
U6O	Maxillary 1st permanent molar occlusal	OP	Occlusal plane
U6D	Maxillary 1st permanent molar distal	SPS	Soft palate superior point
U6M	Maxillary 1st permanent molar mesial	SPI	Soft palate inferior point
L6O	Mandibular 1st permanent molar occlusal	AA A	Anterior pharynx at A
L6D	Mandibular 1st permanent molar distal	PA A	Posterior pharynx at A
L6M	Mandibular 1st permanent molar mesial	AA Mx1	Anterior pharynx at Mx1
L1T	Lower central incisor incisal tip	PA Mx1	Posterior pharynx at Mx1
L1R	Lower central incisor root tip	AA B	Anterior pharynx at B
L1L	Lower central incisor lingual gingival border	PA B	Posterior pharynx space at B
L1G	Lower central incisor labial gingival border	AA P	Anterior pharynx at Pg
U1T	Upper central incisor incisal tip	PA B	Posterior pharynx at Pg
U1R	Upper central incisor root tip	THR	Throat point
U1L	Upper central incisor lingual gingival border	TVL	McNamara true vertical line
U1G	Upper central incisor labial gingival	FH	Frankfort horizontal line

**Table 2:** Measurements and definitions used in the cephalometric analysis

Measurement (unit)	Description
Maxilla/midface	
SNA (°)	Sagittal position of maxilla
ANS-PNS (mm)	Maxillary length
Co-ANS (mm)	Length condylion to ANS
N/S^ANS/PNS (°)	Palatal plane angle
Ba-S-N (°)	Saddle angle
S-N (mm)	Anterior cranial base length
S-Ba (mm)	Posterior cranial base length
Ba-A (mm)	Length basion to A point
Ba-N-A (°)	angle formed from basion to nasion to A point
FH^N/A (°)	Maxillary depth angle
PNS-Ba (mm)	PNS to basion
A-N true vertical (mm)	Length of A to N, sagittal position of the maxilla
OP^FH (°)	Angle formed by occlusal plane to FH
OP^SN (°)	Angle formed by occlusal plane and sella-nasion
Mandible	
SNB (°)	Sagittal position of mandible
Ba-B (mm)	Length basion to B point
Ba-N-B (°)	Angle formed from basion-nasion-B point
FH-N-Po (°)	Mandibular depth angle
Co-Go-Gn (°)	Gonial angle
Co-Go (mm)	Mandibular length
S-Go (mm)	Length from condylion to pogonion
Co-Pg (mm)	Length from sella to gonion
S/N^Go/Gn (°)	Angle form by the gonion/gnathion and nasion/sella
B-N true vertical (mm)	B to N True vertical
Pg-N true vertical (mm)	Pogonion to N True vertical
Y-axis length (mm)	Length Sella to Gnathion
Y-axis angle (°)	Angle formed from ST G and sella-nasion
Inter-maxillary relationship	
ANB (°)	A-N-B angle
Wits appraisal (mm)	Length along the occlusal plane from perpendicular points to A and B
Co-Pog-Co-ANS (mm)	Harvold analysis
Facial height	
TFH (N-Me) (mm)	Total facial height; distance from nasion to menton
UFH (N-ANS) (mm)	Upper facial height; distance from nasion to ANS
LFH (ANS-Me) (mm)	Lower face height; distance from ANS to menton
LFH/TFH (%)	Lower face height to total face height ratio
PFH (S-Go) (mm)	Posterior facial height; distance from S-Go
AFH (N-Pg) (mm)	Anterior facial height, distance from N to Pg
PFH/AFH (%)	Ratio of the posterior facial height and anterior facial height
Hyoid	
MP-H (mm)	Length of hyoid from line perpendicular to the mandibular plan (Go-Me)
H-Rg (mm)	Length of hyoid to retrognathion
H-C3 (mm)	Length of hyoid to C3
H-C3-Rg (mm)	Length of hyoid from line perpendicular to C3 to retrognathion
H-Me-Go/Me (°)	Angle formed from hyoid, menton, and gonion
H-S (mm)	Distance from hyoid to sella
C2 - H FH perp (mm)	Length of C2-hyoid to the horizontal aspect to FH
C2 - H || FH (mm)	Length of C2-hyoid in the vertical aspect to FH
C3 - H FH perp (mm)	Length of C3-hyoid to the horizontal aspect to FH
C3 - H || FH (mm)	Length of C3-hyoid in the vertical aspect to FH
C4 - H FH perp (mm)	Length of C4-hyoid to the horizontal aspect to FH
C4 - H || FH (mm)	Length of C4-hyoid in the vertical aspect to FH
Airway	
Airway at A point (mm)	Pharyngeal airway length at A point
Airway at Mx1 level (mm)	Pharyngeal airway length along the occlusal plane
Airway at B point (mm)	Pharyngeal airway length at B point
Airway at Pog level (mm)	Pharyngeal airway length at the level of the pogonion
Palate	
ANS/PNS^Me/Go (°)	Angle of palate to mandibular plane
SPS-SPI (mm)	Thickness of soft palate
Dental/soft tissue profile	
Mx1-Mx OP (°)	Upper incisor inclination
Mx1-Sn (mm)	Upper incisor tip projection
Md1-Md OP (°)	lower incisor inclination
Md1-Sn (mm)	lower incisor tip projection
Overjet (mm)	Overjet (measurement of U1T to L1T in the horizontal direction)
Overbite (mm)	Overbite (measurement of U1T to L1T in the vertical direction)
Maxillary anterior height (mm)	Subnasale, U1 tip perpendicular to TVL
Sub-Gl (mm)	Subnasale to soft glabella to TVL
STA-TVL (A') (mm)	Soft tissue point A length to TVL
STB-TVL (B') (mm)	Soft Tissue point B length to TVL
STP-TVL (mm)	Soft tissue pogonion length to TVL
STP-THR (mm)	Soft tissue pogonion to throat point

### Reliability assessment

Prior to conduct of the study, two examiners (NC and VA) were calibrated to trace the lateral cephalometric radiographs in the Dolphin Imaging software. A total of 16 lateral cephalometric radiographs were traced by two examiners independently and their inter-examiner reliability was assessed by intra-class correlation coefficients. When discrepancies in landmark identification were observed, a third examiner (RB) independently evaluated the tracings, and corrections were made.

### Control population

Data for neurotypical patients were obtained from the University of Iowa Facial Growth study that was started in 1946 by Meridith and Higley [18, 19]. The original sample included 89 boys and 86 girls who were North American White children of Northern European descent living in Iowa. Participants were enrolled and had the first lateral cephalometric radiographs exposed at 3 years of age. This cohort had lateral cephalometric radiographs exposed quarterly until the age of 5 years and, thereafter, had lateral cephalometric radiographs exposed semiannually until 12 years of age. Lateral cephalometric radiographs were exposed on an annual basis during adolescence and once during adulthood. The normative values, which are used for comparison in the present study, were obtained from 20 male and 15 female Caucasian participants who participated in the Iowa Growth Study from 4 years of age to adulthood. None of these 35 participants had orthodontic treatment, and all had clinically acceptable occlusion and facial harmony. The parameters assessed in this cohort included skeletal anteroposterior relationships, skeletal vertical relationships, dental angular relationships, dental linear relationships, airway measurements, and soft tissue relationships.

### Statistical approach

Descriptive statistics (including mean, standard deviations, and percentile distributions) were used to summarize the cephalometric data. Cephalometric data were summarized by age (<7.5 years, 7.5 to <11.5 years, 11.5 to <15.5 years, and ≥15.5 years), sex, and race (White versus non-White). Both raw and normalized z-scores were computed. One-sample *t* tests were used to test whether mean z-scores differed from zero. The demographic characteristics (age distribution, gender, race, and ethnicity) between those with or without lateral cephalograms among all study participants were compared by Fisher’s exact tests. All statistical tests were two-sided and a *p* value of <0.05 was deemed to be statistically significant. Statistical analyses were computed using SAS software (version 9.4, SAS Institute, Cary, NC).

## Results

### Characteristics of the study population

The characteristics of the study population are summarized in Table 3. A total of 130 participants enrolled in the larger study, among whom a dental examination was completed on 69 participants, and 27 of these patients were able to have a lateral cephalometric radiograph exposed. The final study sample for the present study comprised of 27 patients which included 13 Whites and 9 non-White participants (which included Black [1] participants), Asian [5], and other races [3]). For 5 participants, parents/guardians declined to report race. Sixteen were males, and 11 were females. Ten patients were aged <7.5 years, 11 were 7.5 to <11.5 years, 3 were 11.5 to <15.5 years, and 3 were ≥15.5 years. When comparing those who had and did not have lateral cephalograms among all patients who had a dental examination (*N* = 69), we found no statistically significant difference in distribution of gender. Those aged <7.5 years were less likely to have a lateral cephalogram (*p* < 0.001). Whites were also less likely to have a lateral cephalogram (*p* = 0.04).

**Table 3:** Characteristics of study cohort

Variable	Level	Overall (total *N* = 69)	Cephalograms		*p* value
			Had lateral cephalogram (*N* = 27)	Did not have lateral cephalogram (*N* = 42)	
Age in years	< 7.5 years	63.8 %	37 %	81 %	<0.001
	7.5 to <11.5 years	26.1 %	40.7 %	16.7 %	
	11.5 to <15.5 years	4.3 %	11.1 %	0	
	≥15.5 years	5.8 %	11.1 %	2.4 %	
Gender	Male	62.3 %	59.3 %	64.3 %	0.80
	Female	37.7 %	40.7 %	35.7 %	
Race	White	66.7 %	48.1 %	78.6 %	0.04
	Black	2.9 %	3.7 %	2.4 %	
	Asian	8.7 %	18.5 %	2.4 %	
	Other race	7.2 %	11.1 %	4.8 %	
	Multiracial	1.4 %	0 %	2.4 %	
	Unknown	13 %	18.5 %	9.5 %	

### Reliability assessment

The reliability assessments (intra-examiner and inter-examiner) are summarized in Table 4. A total of 16 lateral cephalograms were re-traced by one examiner for intra-examiner reliability assessment. We found that the intra-rater agreement for maxillary, midface, mandibular, inter-maxillary, facial height, hyoid, airway, palate, dental, and soft tissue profile measurements were high with an intra-class correlation coefficient of 0.80. For a vast majority of these variables, the inter-examiner reliability was also found to be strong. Measurements using PNS were found to be moderate or poor in the initial assessment. Following this, a third examiner independently evaluated all lateral cephalograms, and corrections were made wherever the PNS landmark was identified incorrectly. In the final analysis presented in this paper, the measurements computed from the correct landmarks are presented.

**Table 4:** Intra-examiner and inter-examiner reliability of lateral cephalometric radiograph measurements

Group	Variable	Intra-examiner reliability: ICC	Inter-examiner reliability: ICC
Maxilla/midface	SNA	0.94	0.75
	ANS-PNS (mm)	0.98	0.37
	Co-ANS (mm)	0.99	0.93
	N/S^ANS/PNS palatal plane angle (°)	0.93	0.49
	Ba-S-N (°)	0.97	0.59
	S-N anterior cranial base (mm)	0.98	0.98
	S-Ba posterior cranial base (mm)	0.99	0.95
	Ba-A (mm)	0.99	0.95
	Ba-N-A (°)	0.93	0.81
	FH^N/A maxillary depth (°)	0.86	0.68
	PNS-Ba (mm)	0.97	0.27
	A-N true vertical (mm)	0.90	0.87
	OP^FH (°)	0.94	0.66
	OP^SN (°)	0.97	0.68
Mandible	SNB	0.98	0.76
	Ba-B (mm)	1.00	0.95
	Ba-N-B (°)	0.98	0.82
	FH-N-Po facial angle/mandibular depth angle (°)	0.91	0.78
	Co-Go-Gn gonial angle (°)	0.93	0.83
	Co-Go (mm)	0.84	0.70
	S-Go (mm)	0.94	0.88
	Co-Pog (mm)	0.99	0.97
	S/N^Go/Gn (°)	0.97	0.88
	B-N true vertical (mm)	0.94	0.83
	Pg-N true vertical (mm)	0.95	0.87
	Y-axis length (mm)	0.99	0.98
	Y-axis angle (°)	0.99	0.79
Inter-maxillary relationship	ANB	0.96	0.90
	Wits appraisal (mm)	0.95	0.70
	Co-Pog-Co-ANS	0.99	0.45
Facial height	TFH (N-Me) (mm)	1.00	0.98
	UFH (N-ANS) (mm)	0.99	0.93
	LFH (ANS-Me) (mm)	1.00	0.98
	LFH/TFH (%)	0.99	0.77
	PFH (S-Go) (mm)	0.92	0.82
	AFH (N-Pg) (mm)	1.00	0.98
	PFH/AFH (%)	0.92	0.85
Hyoid	MP-H (mm)	0.99	0.97
	H-Rg (mm)	1.00	0.90
	H-C3 (mm)	0.99	0.93
	H-C3-Rg (mm)	0.99	0.99
	H-Me-Go/Me (°)	0.99	0.94
	H-S (mm)	1.00	0.98
	C2 - H FH perp (mm)	0.97	0.97
	C2 - H || FH (mm)	1.00	0.95
	C3 - H FH perp (mm)	0.96	0.96
	C3 - H || FH (mm)	1.00	0.94
	C4 - H FH perp (mm)	0.92	0.80
	C4 - H || FH (mm)	0.99	0.88
Airway	Nasopharynx airway at A point level (mm)	0.93	0.60
	Oral pharyngeal airway space at Mx1 level (mm)	0.99	0.11
	Hypopharynx airway space at B point level (mm)	0.99	0.03
	Deep pharynx airway at Pog level (mm)	0.99	0.10
Palate	AGSP-PNS (palate length) (mm)	0.97	0.34
	ANS/PNS^Me/Go (angle of palate to MP) (°)	0.95	0.84
	SPS-SPI (max soft palate thickness) (mm)	0.94	0.57
Arnett/Mc modified for OSA	Upper incisor inclination (Mx1-MxOP) (°)	0.86	0.49
	Upper incisor tip projection (Mx1-Sn) (mm)	0.95	0.86
	Lower incisor inclination (Md1-MdOP) (°)	0.94	0.76
	Lower incisor tip projection (Md1-Sn) (mm)	0.96	0.85
	Overjet (mm)	0.91	0.56
	Overbite (mm)	0.79	0.04
	Maxillary anterior height (mm)	0.89	0.65
	Sub-Gl (mm)	−0.07	−0.22
	STA-TVL (A') (mm)	0.87	0.21
	STB-TVL (B') (mm)	0.95	0.80
	STP-TVL (mm)	0.96	0.84
	STP-THR (mm)	0.85	0.21

### Dental examination

The dental examination served three purposes: to evaluate each participant’s soft tissue profile, to evaluate the oropharyngeal airway, and to establish each participant’s occlusion. In this study cohort of 27 patients, none had mandibular hyperplasia and 12 had maxillary hyperplasia. One patient was classified as Friedman grade I, 13 were grade II, and 11 were grade III. None had grade IV tonsils. Six patients were classified as Mallampati class I, 14 had class II, 5 had class III, and 1 patient was classified as class IV.

### Lateral cephalometric radiograph assessment

#### Skeletal maxillary, mandibular, and inter-maxillary measurements

**Fig. 2:**
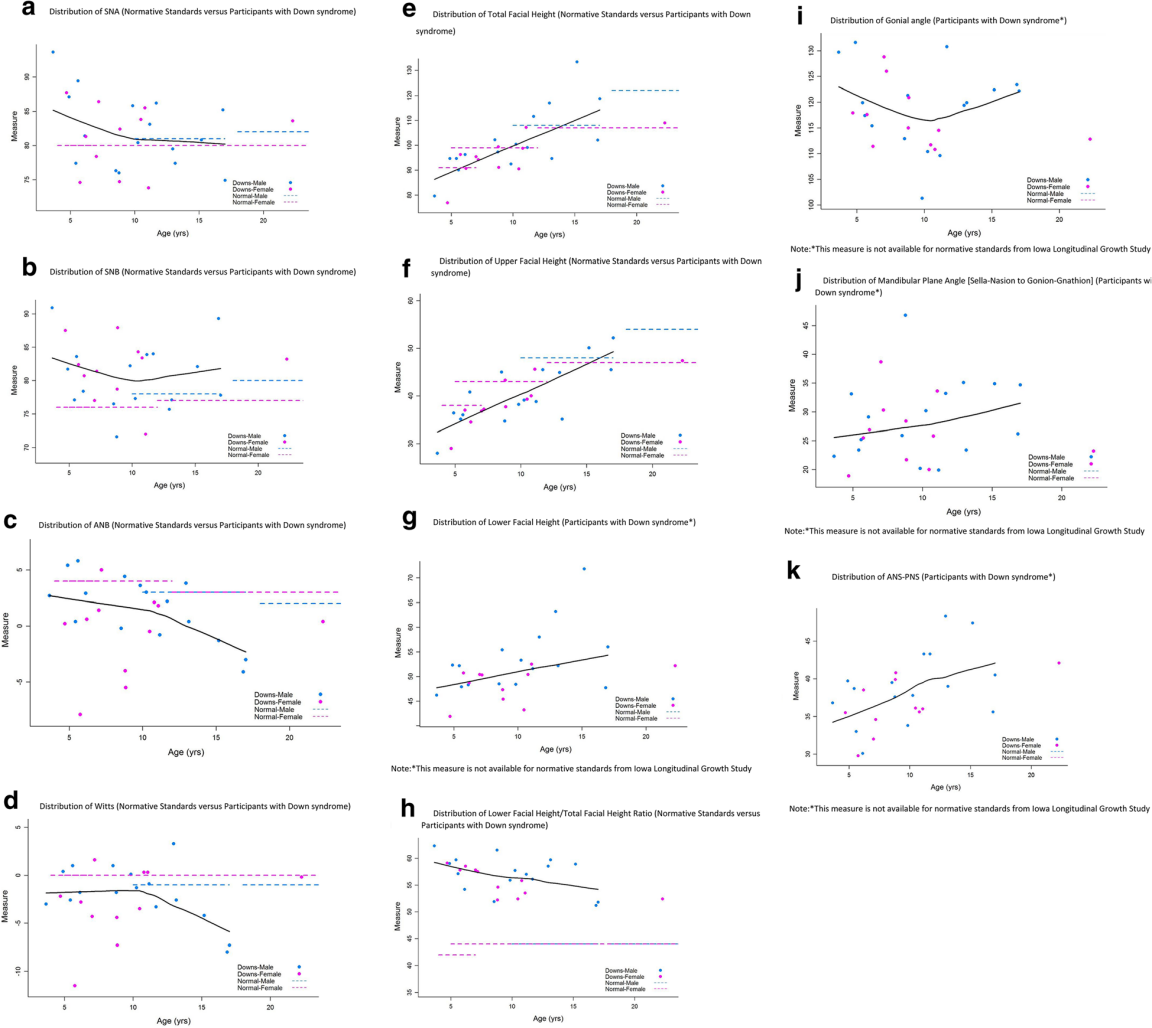
Skeletal maxillary, mandibular, and inter-maxillary measurements. **a** Distribution of SNA (normative standards versus participants with Down syndrome). **b** Distribution of SNB (normative standards versus participants with Down syndrome). **c** Distribution of ANB (normative standards versus participants with Down syndrome). **d** Distribution of Wits (normative standards versus participants with Down syndrome). **e** Distribution of total facial height (normative standards versus participants with Down syndrome). **f** Distribution of upper facial height (normative standards versus participants with Down syndrome). **g** Distribution of lower facial height (participants with Down syndrome*). **h** Distribution of lower facial height to total facial height ratio (normative standards versus participants with Down syndrome). **i** Distribution of gonial angle (participants with Down syndrome*). **j** Distribution of mandibular plane angle [sella-nasion to gonion-gnathion] (participants with Down syndrome*). **k** Distribution of ANS-PNS (participants with Down syndrome*)

The distribution of maxillary and mandibular measurements (SNA, SNB, ANB, Wits, facial height, gonial angle, mandibular plane angle, and ANS-PNS) in this study cohort is presented in Fig. 2a–k. The distributions are presented by age and gender. The majority of SNA and SNB measurements (Fig. 2a, b) were clustered around 80°. SNA tended to be lower among older participants. Compared to normative standards, SNB measurements were higher among female DS participants. The ANB angle was more negative among older participants (Fig. 2c) and lower compared to the normative standards. The ANB angle and Wits measurements (Fig. 2d) suggested that older participants tended to have a class III skeletal pattern. Total facial height (Fig. 2e), upper facial height (Fig. 2f), and lower facial height (Fig. 2g) were all larger among older participants, parallel with normative standards. However, lower facial height to total facial height ratio (Fig. 2h) for the study cohort was notably higher compared to normative standards, with the largest deviation from norms among younger participants. Gonial angle (Fig. 2i) and mandibular plane angle (Fig. 2j) increased with age. The ANS-PNS length (Fig. 2k) was greater among older participants.

#### Overjet, overbite, and incisor angulations

**Fig. 3:**
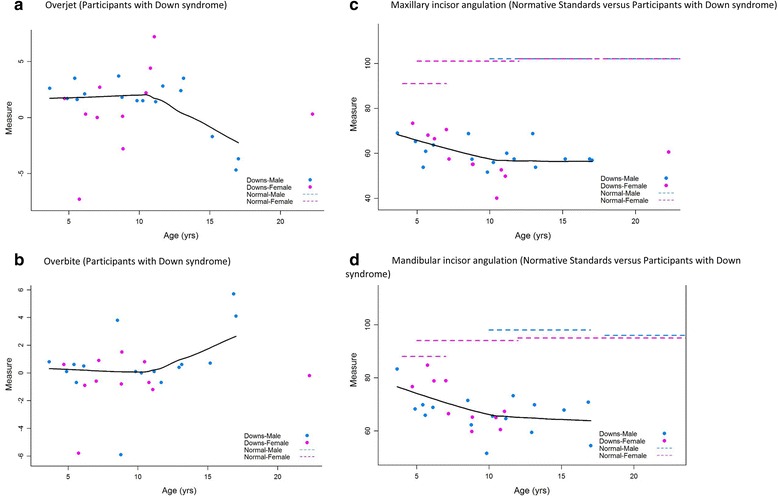
Overjet, overbite, and incisor angulations. **a** Overjet (participants with Down syndrome). **b**. Overbite (participants with Down syndrome). **c**. Maxillary incisor angulation (normative standards versus participants with Down syndrome). **d**. Mandibular incisor angulation (normative standards versus participants with Down syndrome)

Overjet (Fig. 3a) appeared to be heavily clustered between 0 and 5 mm. The majority of patients had an overbite close to zero (Fig. 3b). For the entire study cohort, the maxillary incisor angulation was lower than the normative standards (Fig. 3c). Deviation of the maxillary incisor angulation from normative standards was greater among older participants. The mandibular incisor angulation (Fig. 3d) was also lower than the normative standards with larger deviations among older participants.

#### Airway measurements

**Fig. 4:**
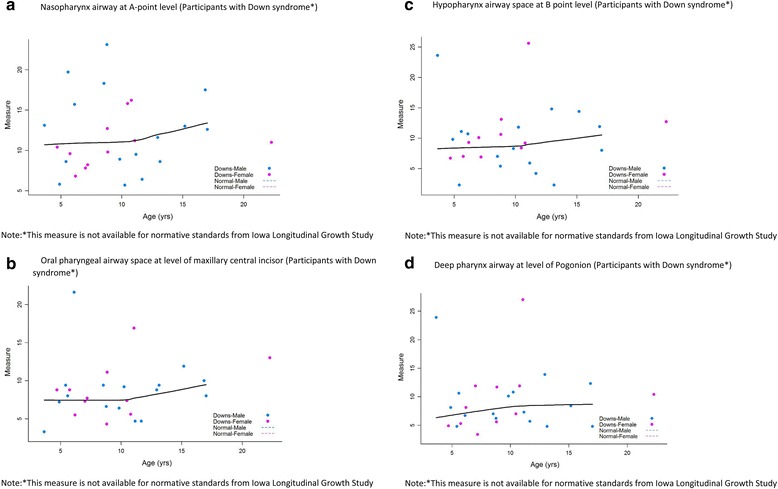
Airway Measurements. **a**. Nasopharynx airway at A point level (participants with Down syndrome*). **b**. Oral pharyngeal airway space at level of maxillary central incisor (participants with Down syndrome*). **c**. Hypopharynx airway space at B point level (participants with Down syndrome*). **d**. Deep pharynx airway at level of pogonion (participants with Down syndrome*)

The nasopharynx airway at A point level (Fig. 4a), oral pharyngeal airway space at the level of maxillary central incisor (Fig. 4b), hypopharynx airway space at B point level (Fig. 4c), and deep pharynx airway at the level of pogonion (Fig. 4d) tended to be larger among older participants.

## Discussion

The present study compared craniofacial features as assessed by lateral cephalometric radiographs in children with DS and neurotypical controls obtained from the Iowa longitudinal growth study. A cross-sectional study design was used to assess differences in craniofacial features across different age groups. Craniofacial lateral cephalometric measurements were also compared by gender. Apart from the primary outcomes (skeletal maxillary/mandibular measurements, overjet, overbite, and airway measurements), our study also documented more than 50 other different craniofacial cephalometric measurements for which no normative standards are currently available (these will be made available to interested readers upon request).

Prior studies examining craniomorphological features in patients with DS have shown that they are more likely to have maxillary hypoplasia, midface retrusion, and skeletal class III pattern [5–12]. Consistent with these prior studies, our study cohort also demonstrated a class III skeletal pattern. This was more pronounced in the older age groups as compared to the younger age groups. Our study cohort also had an increased proportionate lower anterior face height to total facial height compared to normative standards. While prior studies have shown wide variations in mandibular manifestations such as increased or reduced gonial angles and mandibular plane angles, our study results showed that the mandibular plane angle tended to increase with age [5–12]. These are the patients that are likely to develop skeletal anterior open bites as they grow further. Previous studies showed that patients with DS present with proclined maxillary and mandibular anterior teeth [5–12]. Typically, in patients with well-positioned maxillary and mandibular incisors, the maxillary incisors are angled at close to 105° in relation to the SN plane, and mandibular incisors are around 90° to the mandibular plane. Patients in our study cohort had retroclined maxillary and mandibular incisors when compared to normative standards.

Our study findings have important clinical implications for orthodontic treatment planning. As mentioned earlier, patients with DS tended to develop into class III skeletal patterns. The underlying skeletal issues must be thoroughly considered by orthodontists before embarking on a comprehensive phase of orthodontic treatment. In such patients, it would be better to wait until all growth is complete, and frequently, these patients may benefit with orthognathic surgery in conjunction with orthodontic treatment.

These study results and conclusions are subject to a few limitations. First, the present study is a cross-sectional analysis of lateral cephalometric radiograph compared to normative controls. The nature of the study design precludes us from evaluating any cause-and-effect relationship. The published controls were all Caucasian and of North European descent. In contrast, close to 50 % of our study population were non-Caucasian. There is considerable literature demonstrating racial variations in cephalometric normative values. However, one should consider that the present study included participants with DS, and these cohorts present with typical craniofacial features that do not vary much with race. Second, there is a potential for selection bias. In the present study, lateral cephalometric radiographs could be exposed on only 27 out of 130 participants who enrolled in the larger study. The majority of patients with DS who could tolerate taking a lateral cephalometric radiograph were over the age of 7.5 years, and the youngest patient on which a lateral cephalometric radiograph was exposed was 3.3 years old. We found that many of our younger patients were unable to tolerate the relatively lengthy process of being properly positioned and remaining still, which is required for successful exposure of the lateral cephalometric radiograph. If the participant moved during any one point of the process, the radiograph would be deemed unsuccessful. Third, while comparing the cephalometric values between DS and normative controls, we conducted multiple comparisons across age groups and within race and gender, increasing the potential for type 1 errors. Conversely, the final sample size of 27 is small, increasing the potential for type 2 errors and thus to low power for statistical tests. Unfortunately, it is difficult to recruit sufficiently large numbers of patients with DS who can tolerate exposure of lateral cephalometric radiographs and clinical examinations. This is not surprising as children with DS are known to have varying levels of developmental and cognitive delays. Finally, since the present study was conducted at a single center, the external validity and generalizability of the study findings are limited. Notwithstanding, our data contribute a preliminary understanding of this population to the research literature.

## Conclusions

Patients with Down syndrome present typically with class III skeletal pattern and long lower anterior facial heights. In patients with Down syndrome, a comprehensive phase of orthodontic treatment may be best initiated following cessation of growth.
